# Survey dataset on the level of sustainable consumption of Malaysian households from the perspective of income and consumption expenditure

**DOI:** 10.1016/j.dib.2021.106743

**Published:** 2021-01-14

**Authors:** Noorhaslinda Kulub Abd Rashid, Nor Fatimah Che Sulaiman, Zuraini Anang, Bayu Taufiq Possumah, Suriyani Muhamad, Nor Hayati Sa'at, Fauziah Abu Hasan, Hairunnizam Wahid

**Affiliations:** a Faculty of Business, Economics and Social Development, Universiti Malaysia Terengganu, Terengganu, Malaysia; b Institute of Tropical Biodiversity and Sustainable Development, Universiti Malaysia Terengganu, Terengganu, Malaysia; c Center for Sustainable & Inclusive Development (SID), Faculty of Economics & Management, Universiti Kebangsaan Malaysia (UKM), Selangor, Malaysia

**Keywords:** Sustainable consumption, Household, Income, Consumption expenditure

## Abstract

Unsustainable consumption promotes discouraging patterns of consumption with negative impacts on society. It also contributes to inequalities and poverty. Unsustainable patterns of production and consumption undermine development goals in terms of inefficiency and overconsumption. This research explores the level of sustainable consumption of Malaysian households from the perspective of income and consumption expenditure. The analysis is based on cross-sectional data obtained from questionnaires distributed to 635 Malaysian households in eight districts in Terengganu (urban and rural areas) using stratified random sampling. The findings show that the level of sustainable consumption expenditure of Muslim households in Terengganu is still low. Achieving sustainable consumption patterns is more technically and politically complex than changing production patterns because it raises important issues such as human values, equity and lifestyle choices.

## Specifications Table

**Table:** 

Subject	Economics
Specific subject area	Economic Development
Type of data	Table Figure Text
How data were acquired	Survey questionnaire (provided as supplementary file)
Data format	Raw Analysed Descriptive Statistical
Parameters for data collection	Sustainable consumption, income and consumption expenditure
Description of data collection	The data is based on cross-sectional data obtained from questionnaires distributed to 635 Malaysian households using stratified random sampling. The data was screened through normality test and reliability test.
Data source location	Households in eight districts in Terengganu (urban and rural areas) such as Kuala Terengganu, Dungun, Kemaman, Kuala Nerus, Marang, Setiu, Besut, Hulu Terengganu.
Data accessibility	All the data are in this data article as a supplementary data file. Supplementary material associated with this article can be found, in the online version, at http://dx.doi.org/10.17632/snw2pbpbn2.1.

## Value of the Data

• The data will be useful to identify the level of sustainable consumption among Malaysian households in terms of income and expenditure consumption.
• The data is valuable to highlight important issues such as human values, equity and lifestyle choices in order to achieve sustainable consumption.
• Policymakers, stakeholders, governments and researchers can use the data to design programmes while avoiding wasteful spending and negative externalities. In line with government industrial policies, these efforts will commercialise rural-based activities towards market needs, economies of scale and value chain integration.

## 1 Data Description

The data presented here are table and figure formats. Contents include gender, age, marital status, employment, education, health, income, saving and lastly sustainable consumption index (see ‘DIB Dataset.xlsx’ - supplementary files). These data are sorted out from questionnaires. Using these data, we can analyse general statistical characteristics of eight variables, correlation among them and estimate the results of OLS in our research.

Sustainable consumption is a driving force of the global economy in promoting sustainable household lifestyles. This is embodied by the Sustainable Development Goals (SDGs) Goal 12 ‘Responsible consumption and production’ that stated that ‘By 2030, ensure that people everywhere have the relevant information and awareness for sustainable development and lifestyles in harmony with nature’ (UNDP, 2019) [1].

The household expenditure pattern was analysed and presented alongside the Report on Household Expenditure Survey, which covers the nine main groups of goods and services. It is classified based on the ‘Classification of Individual Consumption According to Purpose’ (COICOP) published by the United Nations Statistics Division (UNSD) (DOSM, 2016) [2].

The questionnaire was divided into several sections. Section A contains the characteristics on demographics; Section B refers to the profile of family members; Section C refers to information on sources of income of the household head; Section D contains questions on consumption allocation by categories; Section E concerns savings; Section F regarding the information on household consumption patterns; lastly Section G concerns the quality of life among the households.

Based on the questionnaire, demographic profile data is tabulated. It presents a comprehensive table of respondents by gender, age, education level, number of dependents and income level. Table 1 shows that 523 (82.4%) are male while 112 (17.6%) are female. As for marital status, 77.2% (490) are married and 5.4% (34) are single. 17 of the respondents (2.68%) are in the age group between 20–30 years, 100 of the respondents (15.8%) in the age group between 31–40 years, 131 of the respondents (20.6%) in the age group between 41–50 years, 144 of the respondents (22.7%) in the age group between 51–60 years, 137 of the respondents (21.6%) in the age group between 61–70 years, 92 of the respondents (14.5%) in the age group between 71–80 years, and 14 of the respondents (2.0%) in the age group between 81–90. The majority of the 285 of the respondents (44.9%) have Form Three Assessment which is *Penilaian Menengah Rendah* (PMR) or *Sijil Rendah Pelajaran* (SRP) or equivalent as the highest achievements of their education, followed by 219 of the respondents (34.5%) who have Malaysian Certificate of Education (SPM) or equivalent. Meanwhile, 39 of the respondents (6.1%) have no education background, followed by 36 of the respondents (5.6%) who have Malaysian Higher School Certificate (STPM) or diploma, skill certificates or equivalent and 27 of the respondents (4.3%) who have bachelor's degree. As for the number of dependents, Table 1 indicates that 24 of the respondents (3.8%) have no dependents, 292 of the respondents (46.0%) have 1 to 3 dependents, 256 of the respondents (40.3%) have 4 to six dependents, 57 of the respondents (8.9%) have 7 to 9 dependents, and 6 of the respondents (1.0%) have 10 dependents and above.

**Table 1:** Profile of respondent (N=635).

Respondent's Profile	Frequency	Percentage
Gender (Head of Household)		
Male	523	82.4
Female	112	17.6
Marital status		
Single	34	5.4
Married	490	77.2
Widows/widowers	111	17.5
Age		
20–30	17	2.68
31–40	100	15.8
41–50	131	20.6
51–60	144	22.7
61–70	137	21.6
71–80	92	14.5
81–90	14	2.0
Education level		
Non-schooling	39	6.1
PMR/SRP and below	285	44.9
SPM	219	34.5
STPM/Diploma/Skills Certificates	36	5.6
Bachelor and above	27	4.3
Dependents		
No dependents	24	3.8
1–3 dependents	292	46.0
4–6 dependents	256	40.3
7–9 dependents	57	8.9
10 dependents and above	6	1.0
Income		
RM2000 and below	72	11.3
RM2001-RM4000	305	48.0
RM4001-RM6000	138	21.7
RM6001-RM8000	49	7.7
RM8001-RM10000	22	3.5
RM10001 above	49	7.7

The majority of the 305 of the respondents (48.0%) earned RM2000 to RM4000 per month, followed by 138 of respondents (21.7%) earned RM4001 to RM6000 per month. 11.3% or 72 respondents received RM2000 and below per month, respectively, 49 of respondents (7.7%) earned RM6001 to RM8000 and RM10001 and above per month. Meanwhile, 22 of respondents (3.5%) earned RM8001 to RM10000 per month.

The level of sustainable consumption among Malaysian Muslim household was derived from the formation of the ‘Sustainable Consumption Index’ which consist of consumption expenditure, household behaviour, and religiosity dimension. The instrument for index formation was developed based on past literature, expert opinion and research objectives. Such index measurements have been used in previous studies (see Hairunnizam et al. [3]; Rahmah et al., 2010 [4]).

**Fig. 1:**
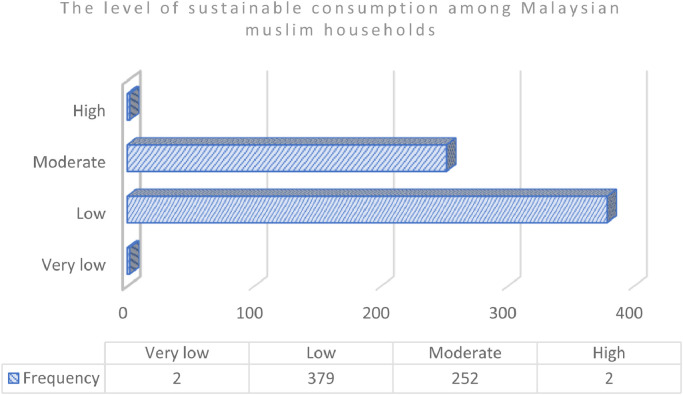
The level of sustainable consumption among Malaysian Muslim households.

Table 2 provides the scale of Sustainable Consumption Index with five categories. Based on Fig. 1, the level of sustainable consumption among Malaysian Muslim households can be classified from ‘High’ to ‘Very Low’ scales since there was no score value among the respondents. These ranking categories scale takes into account three indicators such as consumption expenditure, household behaviour and religiosity. The consumption expenditure dimension elaborates on nine components of consumption expenditures of households. On the other hand, the household behaviour dimension explains the household lifestyle, sources information and shopping behaviour. The religiosity dimension includes aspects of aqidah (faith and belief), syariat (practices and activities) and akhlak (moralities and ethic) deeds.

**Table 2:** The Scale of Sustainable Consumption Index.

Index value	Scale
Less than 0.2000	Very low
0.2001 to 0.4000	Low
0.4001 to 0.6000	Moderate
0.6001 to 0.8000	High
More than 0.8001	Very high

The formation of a sustainable consumption index was done using a formula adapted from a study by Rahmah et al. in their study to measure the competitiveness of workers in the education sector. The index formation consists with many steps as mentioned below. Eq. (1) is used to obtain the index for each dimension.

(1) $$I_{ij}=\;\frac{1}{l_{k}}\sum^{{\tilde{X}}_{ik}}\;$$

Whereby $I_{ij}$is the index of the *j* indicator for the individual *i* obtained on average after dividing by the number of items ($l_{k}$) found in each of the *j* indicators. Next, ${\tilde{X}}_{ik}$ is the normalization of the total value of the individual score *i* for each item *k* calculated using the formula (2):

(2) $${\tilde{X}}_{i}=\;\frac{Actual\;value_{k}-Minimum\;value_{k}}{Maximum\;value_{k}-Minimum\;value_{k}}\;$$

Eq. (3) showed the index of the *j* component obtained through the above equation, while *m* is the number of components found in the *y*-dimension. While *Wj* is a weighted value according to their respective interests as mentioned above.

(3) $$Z_{iy}=\;\sum_{j=1}^{m}w_{j}I_{ij\;}\;$$

Next, the formation of the Sustainable Consumption Index will be done as a final step by summing the consumption expenditure, household behaviour and religiosity, which is derived from Eq. (4):

(4) $$Sustainable\;Consumption\;Index\;{\left(SCI\right)}_{i}\;=\;\sum_{y}w_{y}Z_{iy}$$

The index will be at a value of 0 to 1 with a value approaching 1 indicating that the level of sustainable consumption is at a very good level while the value approaching 0 is the opposite. The index scale can be summarized as in Table 2.

Meanwhile, the survey questionnaire used a four-point Likert scale for simplification and to collect specific responses from respondents without misleading the respondents. This four-point likert scale has been practised by previous studies such as Tawalbeh [5] and Hashim and Yunus [6], among others. In addition, this four-point likert scales represent question items related to consumption expenditures such as food, apparel, transportation, residential, health, education, recreation, loans and communication, with a score value 1 to 4 (never, rarely, frequent and very frequent) as suggested by Maraolo et al. [7].

Based on Fig. 1, the highest score of the level of sustainable consumption among Malaysian Muslim households are low with 379 number of respondents (59.7%), followed by moderate (39.7%) with 252 respondents and 2 for very low and high with 0.31% respectively.

Table 3 shows the factors contributing to sustainable consumption among Malaysian Muslim households. Multiple regression methods are used in estimating the sustainable consumption (as independent variables) as well as gender, age, employment, education, health, marital status, income and saving (as dependent variables). With multiple regression the relationship is described using a variation of the equation of a straight line.

**Table 3:** Factors influencing sustainable consumption among Muslim households.

	Unstandardised Coefficients		Standardised Coefficients		
	B	Std. Error	Beta	t	Sig.
(Constant)	.400	.033		12.170	.000
Gender	–.052	.009	–.242	–5.655	.000⁎⁎⁎
Age	.000	.000	–.063	–1.439	.151
Employment	.005	.003	–.075	–1.867	.062*
Education	.007	.002	.141	3.558	.000⁎⁎⁎
Health	.006	.005	.051	1.291	.197
Marital status	.022	.008	.122	2.882	.004⁎⁎
Income	.029	.005	.225	6.216	.000⁎⁎⁎
Saving	–.019	.003	–.235	–6.524	.000⁎⁎⁎

⁎ Significant level at 0.10.

⁎⁎ Significant level at 0.05.

⁎⁎⁎ Significant level at 0.01.

The multiple regression equation explained above takes the following form:

$$y=\;\beta_{1}X_{1}+\beta_{2}X_{2}+\;\ldots \ldots +\beta_{8}X_{8}+\in$$

Here, $\beta_{i}$’s (i=1, 2 … 8) are the regression coefficients, which represent the value at which the criterion variable changes when the predictor variable changes. The beta value is used in measuring how effectively the predictor variable influences the criterion variable, it is measured in terms of standard deviation.

## 2 Experimental Design, Materials and Methods

The researcher adopted a survey research design to obtain data from 635 Malaysian households in eight districts in Terengganu (urban and rural areas). Data were gathered by means of a structured questionnaire (Appendix 1). The data were qualitatively analysed and presented in Tables 1, 2, 3 and Fig. 1.

## Ethics Statement

Ethical consideration in the research process was ensured because administering the questionnaires to respondents was based on their willingness to respond to the research instrument. This survey was approved by the Ethics Committee of Universiti Malaysia Terengganu & Kuala Terengganu District, Malaysia, with reference No. PDKT 220/65 Jld 4 (5). This study confirms that consent was obtained from individuals who participated in the survey.

## CRediT Author Statement

**Noorhaslinda Kulub Abd Rashid:** Conceptualization, Methodology; **Nor Fatimah Che Sulaiman & Zuraini Anang:** Formal analysis; **Bayu Taufiq Possumah & Nor Hayati Sa'at:** Writing-Original draft; **Suriyani Muhamad:** Writing-Review & Editing; **Fauziah Abu Hasan & Hairunnizam Wahid:** Supervision.

## Declaration of Competing Interest

The authors declare that they have no known competing for financial interests or personal relationships that could have appeared to influence the work reported in this paper.
